# Surgical nurses’ perceptions of experienced sexual harassment behaviors

**DOI:** 10.1186/s12912-025-04202-6

**Published:** 2025-12-09

**Authors:** Dilek Aygin, Cansu Kubilay, Büşra Ecem Kumru, Hilal Kaynak Aydoğmuş

**Affiliations:** 1 Department of Nursing, Faculty of Health Sciences, Sakarya University, Sakarya, Türkiye; 2 Department of Nursing, Institute of Health Sciences, Sakarya University, Sakarya, Türkiye; 3 Home Patient Care Programme, Osmaneli Vocational School, Bilecik Şeyh Edebali University, Bilecik, Türkiye; 4 Home Patient Care Programme, Vocational School of Health Services, Üsküdar University, Istanbul, Türkiye

**Keywords:** Surgical nursing, Sexual harassment, Workplace violence, Occupational health, Health personnel

## Abstract

**Background:**

Nurses who are in continuous interaction with patients and their relatives may occasionally be exposed to harassing behaviors. Surgical nurses, who often have prolonged patient contact due to the shift system, constitute a significant proportion of the overall nursing workforce. This study aimed to identify the sexual harassment behaviors experienced by surgical nurses working in Türkiye, the perceived severity of these behaviors, and the related factors.

**Methods:**

This descriptive cross-sectional study was conducted with 302 surgical nurses between March 28, 2023, and February 28, 2024. Data were collected via an online questionnaire distributed through social media platforms using Google Forms. The instruments included a Descriptive Characteristics Form, the Sexual Harassment Behaviors of Care Workers by Patients or Clients Scale (12 items; verbal, observed, and physical subscales), and an Attitudes and Behaviors Toward Sexual Harassment Form. The study was approved by the Üsküdar University Non-Interventional Research Ethics Committee (Decision No: 03, Approval Date: 28 March 2023). Non-parametric tests (Mann–Whitney U and Kruskal–Wallis) were performed, with significance set at *p* < 0.05.

**Results:**

Among surgical nurses, 53.6% reported previous exposure to violence, and 48.1% reported sexual violence. The most frequently identified perpetrators were patients and their relatives (59.3%). While 40.7% of nurses reported harassment to a manager, only 32.3% stated that legal action was taken. Female nurses had significantly higher overall perception scores than males (*p* = 0.010). Perception scores were also significantly associated with education level, marital status, prior exposure to violence or sexual violence, and perpetrator identity (*p* < 0.05). Subscale analyses showed consistent patterns across verbal, observed, and physical harassment dimensions.

**Conclusions:**

Sexual harassment remains a critical issue among surgical nurses and is often underreported. Institutional support mechanisms, training, and confidential reporting systems are essential to ensure workplace safety and protect nurses’ well-being.

**Trial registration:**

Not applicable (observational study; no healthcare intervention on human participants).

**Supplementary Information:**

The online version contains supplementary material available at 10.1186/s12912-025-04202-6.

## Background

Workplace violence is a global public health concern that warrants urgent, coordinated policy action [1]. Defined as behaviors that inflict physical or psychological harm, it includes physical, verbal, psychological, and sexual forms [2–6]. Within this spectrum, sexual violence often involving physical assault encompasses unwanted and offensive sexual behaviors expressed verbally, nonverbally, or visually [3]. Workplace sexual harassment (WSH) undermines employee health and safety and erodes organizational productivity and reputation. Evidence links WSH to heightened psychological morbidity among victims including depressive symptoms, post-traumatic stress responses, anxiety, and fear alongside physical sequelae such as hypertension and sleep disturbances; cumulatively, absenteeism, presenteeism, organizational withdrawal, and labor loss drive measurable productivity declines [4, 7].

Nurses face a disproportionate risk of WSH relative to other occupations, a pattern attributed to frequent, often unavoidable physical contact and the intimate nature of caregiving [5, 8]. Those working in surgical settings may be especially exposed owing to social, cultural, and institutional dynamics; perpetrators include patients, family members, and colleagues [9, 10]. Exposure is associated with serious psychological consequences and decrements in quality of life and care [11]. Prevalence estimates suggest nurses experience sexual harassment far more frequently than other professional groups, with reports indicating up to a sixteen-fold difference [12]. Meta-analytic data further indicate a 12-month prevalence of 12.6% and a lifetime prevalence of 53.4% among clinical nurses [13]. In a recent multicenter study of operating room nurses, 63% reported sexual harassment in the prior year; gender-based harassment was most common (56.7%), followed by unwanted sexual attention (28.6%) and sexual coercion (13.6%), with surgeons identified as the primary perpetrators (81.2%) [14].

In Türkiye, pioneering work by Çelik documented that 37.1% of nurses reported experiencing sexual harassment, most frequently perpetrated by physicians, with a notable proportion indicating passive coping (e.g., “did nothing”) [5]. Subsequent studies expanded the scope to patient-initiated behaviors, yielding single-center prevalence estimates as high as 71.7% among nurses, while multi-site surveys of healthcare workers identified substantial overall workplace-violence exposure that included a sexual-harassment component (e.g., 6.4%) [15, 16]. More recent national and regional analyses similarly suggest that approximately one in two healthcare personnel encounter workplace violence at least once, underscoring sexual harassment as a persistent, system-level problem within the healthcare setting [17].

The mental-health burden of WSH is pronounced: victims particularly when the perpetrator is a supervisor report greater psychological distress and reduced job satisfaction, reinforcing vulnerability and powerlessness [18, 19]. These findings align with broader evidence on the harms of workplace violence in healthcare [20, 21]. Despite the elevated risk among surgical nurses, studies in Türkiye that specifically examine perceptions of patient- or family-initiated sexual harassment remain limited, and much of the existing literature targets general healthcare staff rather than surgical units using validated measures. In Türkiye, studies investigating sexual harassment among surgical nurses that address both exposure and attitudinal–behavioral dimensions through validated measurement tools remain limited. This study seeks to fill this gap by providing a comprehensive assessment of surgical nurses’ perceptions, attitudes, and responses to sexual harassment, thereby contributing empirical evidence to guide preventive strategies and institutional policy development in healthcare settings. Accordingly, this study aims to characterize surgical nurses’ perceptions of experienced sexual harassment behaviors and identify factors shaping these perceptions, to inform preventive institutional strategies, strengthen managerial and peer support, and promote safer, more ethical surgical work environments.

## Materials and methods

### Study design

This study employed a descriptive cross-sectional design.

#### Study population and sample

The study population consisted of nurses working in surgical units across hospitals in Türkiye. The required sample size was calculated using the G*Power 3.1.9.7 program with an effect size (f) of 0.30, statistical power (1–β) of 0.95, and a significance level (α) of 0.05. Based on power analysis parameters, the minimum required number of participants was calculated as 272 surgical nurses. Considering potential non-response and data loss, a total of 302 nurses were included in the final analysis. Participants were selected using a convenience sampling method. The study invitation was distributed through professional nursing networks and social media platforms to reach nurses working in surgical units across different hospitals in Türkiye. Nurses who met the inclusion criteria being actively employed in a surgical clinic and voluntarily agreeing to participate were included in the study. Data collection continued until the predetermined sample size was reached.

#### Data collection tools

Data were collected using the Introductory Characteristics Form, the Sexual Harassment Behaviors of Care Workers by Patients or Clients Scale, and the Attitudes Toward Sexual Harassment Form.

1. **Introductory characteristics form**: The Descriptive Characteristics Form was developed by the researchers in line with the literature review and consisted of eight questions to determine the sociodemographic and occupational characteristics of the nurses (gender, age, education level, marital status, number of children, unit of employment, working time in the profession, and daily working hours) (Refer to Supplementary File 1 for the English version of the form).
2. **Sexual harassment behaviors of care workers by patients or clients scale**: This scale, originally developed by Vincent-Höper et al., measures sexual harassment behaviors directed at healthcare workers [22]. The Turkish adaptation and validation of the scale were conducted by Ayar et al. [23]. The scale comprises 12 items and includes three subdimensions: verbal sexual harassment (6 items), observed sexual harassment (2 items), and physical sexual harassment (4 items).
  The items are rated on a 6-point Likert scale ranging from 1 (never) to 6 (almost daily), with higher scores indicating a higher frequency of sexual harassment experiences. All items are positively scored, and there are no reverse-coded items.
  In the Turkish validation study, the Cronbach’s alpha coefficients were 0.90 for verbal sexual harassment, 0.81 for observed sexual harassment, 0.77 for physical sexual harassment, and 0.91 for the total scale, indicating high internal consistency.
3. **Attitudes and behaviours towards sexual harassment form**: This form was developed by the researchers based on a literature review. It consists of 10 items evaluating the actions taken by nurses when exposed to different types of sexual harassment. Items assess whether nurses report incidents to a manager, initiate legal action, receive support from managers or colleagues, seek psychological help, or report the incident to law enforcement, as well as by whom the harassment was perpetrated (Refer to Supplementary File 2 for the English version of the form.) Data were collected between 28 March 2023 and 28 February 2024 using an online questionnaire created via Google Forms. The survey link was distributed through social media platforms including WhatsApp, Twitter, and Instagram. The data were analyzed using appropriate statistical methods.

### Statistical analysis

#### Objective 1

To determine nurses’ exposure levels to sexual harassment and their descriptive characteristics.

- **Variables**: Demographic and professional characteristics (age, gender, marital status, education, years of experience, etc.)
- **Analysis**: Descriptive statistics — categorical variables were summarized as frequencies and percentages; continuous variables as mean, standard deviation, median, minimum, and maximum.

#### Objective 2

To examine whether exposure to sexual harassment differed according to nurses’ demographic and professional characteristics.

- **Variables**: Exposure scores (scale subdimensions) and demographic/professional variables.
- **Analysis**:
  - Mann–Whitney U test for comparisons between two independent groups,
  - Kruskal–Wallis H test for comparisons among more than two groups.

#### Objective 3

To analyze nurses’ reporting and help-seeking behaviors following sexual harassment.

- **Variables**: Reporting (to manager, to law enforcement), receiving support, psychological help, etc.
- **Analysis**: Descriptive statistics (frequencies and percentages) and Chi-square tests for associations between categorical variables.

### Additional information

The Kolmogorov–Smirnov test was used to assess data normality. A p-value < 0.05 was considered statistically significant.

### Ethics approval and consent to participate

Ethical approval was obtained from the Non-Interventional Research Ethics Committee of Üsküdar University (Approval Date: 28 March 2023; Decision No: 61351342/020-1105). Permission to use the scale was granted by the original authors via e-mail. This study was conducted in accordance with the principles of research and publication ethics. Each participant gave their consent to participate.

## Results

The surgical nurses (*n* = 302) were aged between 20 and 47 years (mean = 31; median = 30); 75.2% were female and 53.0% were married. The average number of children was two. Most surgical nurses (90.7%) held at least a bachelor’s degree. Years of professional experience ranged from 0.3 to 28 years (mean = 7; median = 6), and the mean daily working time was 14.7 h (Table 1).

**Table 1:** Distribution of demographic characteristics of healthcare workers

Variables (*N* = 302)	*n* (%)	Mean ± SD	Median (Min-Max)
Age		31 ± 5	30 (20–47)
Gender			
Male	75 (24.8)		
Female	227 (75.2)		
Education Level			
High School/Associate Degree	28 (9.3)		
Bachelor’s Degree	208 (68.9)		
Master’s Degree	57 (18.9)		
Doctorate	9 (3)		
Marital Status			
Single	142 (47)		
Married	160 (53)		
Number of Children	*n* = 167	2 ± 1	1 (1–3)
Unit You Work			
Emergency Department	5 (1.7)		
Family Medicine	1 (0.3)		
Operating Room	23 (7.6)		
Anesthesia ICU	3 (1)		
Neurosurgery	2 (0.7)		
Surgical Service	6 (2)		
Surgical Intensive Care	5 (1.7)		
Pediatric Surgery ICU	1 (0.3)		
Pediatric Surgery	21 (7)		
Pediatric ICU	1 (0.3)		
Adult ICU	1 (0.3)		
Gastroenterology	6 (2)		
Thoracic Surgery Service	48 (15.9)		
Chest Diseases Service	1 (0.3)		
Eye	5 (1.7)		
Hematology	1 (0.3)		
Gynecology and Obstetrics	27 (8.9)		
Cardiovascular Surgery Service	4 (1.3)		
Cardiovaculer ICU	1 (0.3)		
Cardiology	3 (1)		
Mixed Surgery	1 (0.3)		
ENT Service	13 (4.3)		
Chemotherapy	1 (0.3)		
Nephrology	1 (0.3)		
Neurology ICU	2 (0.7)		
Organ Transplantation	1 (0.3)		
Orthopedic Surgery Service	1 (0.3)		
Orthopedic Service	31 (10.3)		
Palliative ICU	1 (0.3)		
Plastic Surgery	1 (0.3)		
Urology	4 (1.3)		
Burn Unit	1 (0.3)		
ICU	76 (25.2)		
Neonatal ICU	3 (1)		
Professional Working Time (Years)		7.1 ± 5.3	6 (0.3–28)
Daily Working Hours		14.7 ± 6.6	14 (8–24)

Percentages were calculated based on the total sample size (*N* = 302). Values are presented as frequency (n) and percentage (%). Values are presented as Mean ± SD and Median (Min–Max). SD: Standard Deviation; Min: Minimum; Max: Maximum

According to responses on the Sexual Harassment Behaviors of Care Workers by Patients or Clients Scale, 53.6% of surgical nurses reported previous exposure to violence. Among them, 48.1% experienced sexual violence, 32.7% physical violence, 38.3% psychological violence, and 1.9% economic violence. The perpetrators were reported as patients and their relatives (59.3%), managers (22.2%), and colleagues (20.4%).

Regarding workplace sexual harassment, 40.7% of the surgical nurses reported experiencing manager-perpetrated violence, which was predominantly verbal (61.0%) or written (30.1%). Among those who reported such incidents, 32.3% stated that legal action was initiated by their managers. Only 20.2% reported receiving managerial support, while 80.7% received support from their peers. Despite this, 50.8% of surgical nurses reported feelings of isolation following the incident, 24.0% received post-incident psychological support, and 29.3% had contact with law enforcement authorities (Table 2).

**Table 2:** Distribution of nurses’ exposure to violence and reporting behaviors

Variables (*N* = 302)	*n* (%)*
**Previous exposure to violence**	162 (53.6)
Sexual	78 (48.1)
Physical	53 (32.7)
Psychological	62 (38.3)
Economic	3 (1.9)
**Violence perpetrator**	
Parent/Sibling	23 (14.2)
Spouse/Partner	18 (11.1)
Relatives/Friends	17 (10.5)
Colleague	33 (20.4)
Patient or Relative	96 (59.3)
Administrator	36 (22.2)
**Reporting sexual harassment at the workplace to the manager**	123 (40.7)
Verbal notification	75 (61)
Notified in writing	37 (30.1)
I did not report it	24 (19.5)
**If a notification has been made**,** the manager initiates legal proceedings**	32 (32.3)
Support from the manager	
He didn’t provide any support	31 (31.3)
Supported	20 (20.2)
Psychological support	41 (41.4)
Effectively followed the legal process	18 (18.2)
Supported me to move away from the harassing environment/person	13 (13.1)
**Support from your teammates**	88 (80.7)
**Feeling lonely in this process**	61 (50.8)
**Receiving psychological support after the process**	29 (24)
**Applying to the relevant law enforcement authorities/judiciary**	34 (29.3)

Values are presented as frequency (n) and percentage (%). Percentages were calculated based on the total sample size (*N* = 302)

According to Table 3, gender, education level, marital status, prior exposure to violence, experience of sexual violence, and exposure to violence by individuals such as family members, friends, colleagues, and patients or their relatives were significantly associated with the total scores on the Sexual Harassment Behaviors of Care Workers by Patients or Clients Scale (*p* < 0.05).

**Table 3:** Comparison of sexual harassment perception scores by participant characteristics

Descriptive Characteristics and Questions on Violence	Total Score		*p*-value
	Mean ± SD	Median (Min-Max)	
**Gender**			**0.010**
Male	**15.8 ± 4.7**	**13 (12–31)**	
Woman	**18.3 ± 6.9**	**17 (12–43)**	
**Education level**			**0.036**
High School/Associate Degree	**19.4 ± 6.7**	**20 (12–37)**	
Licence	**17 ± 6.1**	**14 (12–43)**	
Master’s degree	**19.1 ± 7.1**	**19 (12–35)**	
PhD	**18.8 ± 8.2**	**14 (12–36)**	
**Marital status**			**0.031**
Single	**17.1 ± 6.9**	**13 (12–42)**	
Married	**18.2 ± 6.1**	**18 (12–43)**	
**Previous exposure to violence**			**< 0.001**
Not exposed to violence	**14.1 ± 4.3**	**12 (12–34)**	
Subjected to violence	**20.8 ± 6.5**	**21 (12–43)**	
**Sexual violence**			**< 0.001**
Not exposed to violence	**17.5 ± 6.1**	**16 (12–42)**	
Subjected to violence	**24.3 ± 4.9**	**24 (12–43)**	
**Physical violence**			**0.002**
Not exposed to violence	**21.9 ± 6.2**	**22 (12–43)**	
Subjected to violence	**18.6 ± 6.6**	**17 (12–36)**	
**Psychological violence**			0.171
Not exposed to violence	21.3 ± 6.3	21.5 (12–43)	
Subjected to violence	20 ± 6.8	19 (12–37)	
**Economic violence**			0.365
Not exposed to violence	20.7 ± 6.4	21 (12–43)	
Subjected to violence	25.3 ± 12.2	28 (12–36)	
**Perpetrator - Family (mother/father/sibling)**			**< 0.001**
Not exposed to violence	**21.6 ± 6.4**	**21 (12–43)**	
Subjected to violence	**16.1 ± 5.1**	**13 (12–27)**	
**Perpetrator - Spouse/Partner**			0.428
Not exposed to violence	20.7 ± 6.4	21 (12–43)	
Subjected to violence	21.9 ± 7.2	21 (12–35)	
**Perpetrator - Relative/Friend**			**0.034**
Not exposed to violence	**20.4 ± 6**	**20 (12–43)**	
Subjected to violence	**24.6 ± 9.3**	**26 (12–42)**	
**Violent offender - Co-worker**			0.059
Not exposed to violence	20.3 ± 6.3	20 (12–43)	
Subjected to violence	22.5 ± 7	22 (12–37)	
**Perpetrator of violence - Patient/Patient Relative**			**0.003**
Not exposed to violence	**18.9 ± 7.1**	**17 (12–42)**	
Subjected to violence	**22.1 ± 5.7**	**22 (12–43)**	
**Violent offender - Manager**			0.711
Not exposed to violence	20.9 ± 6.4	21 (12–43)	
Subjected to violence	20.4 ± 7.1	19 (12–36)	
**Verbal reporting of violence**			0.222
Did not report	22.5 ± 6.1	23 (12–42)	
Reported	21.7 ± 6.2	21 (12–43)	
**Reporting violence in writing**			0.501
Did not report	21.8 ± 5.8	21 (12–43)	
Reported	22.4 ± 6.9	23 (12–42)	
**Failure to report violence**			0.640
Did not report	22 ± 6.4	22 (12–43)	
Reported	21.9 ± 4.7	22 (12–31)	
**The legal process initiated by the manager after the notification**			0.113
No.	21.6 ± 6.8	20 (12–43)	
Yes	22.8 ± 5.7	23 (12–37)	
**Lack of support from the manager**			0.468
No.	22.2 ± 6.2	22 (12–42)	
Yes	21.6 ± 6.9	21 (12–43)	
**Provision of support by the manager**			0.079
No.	22.6 ± 6.6	22 (12–43)	
Yes	19.5 ± 5.2	20 (12–28)	
**Provision of psychological support by the manager**			0.557
No.	21.6 ± 6.6	21.5 (12–43)	
Yes	22.6 ± 6.3	22 (12–42)	
**The status of the manager’s following the legal process**			0.064
No.	21.6 ± 6.5	21 (12–43)	
Yes	24.1 ± 5.9	23 (12–37)	
**Support from the manager to remove from the harassment environment**			0.078
No.	21.5 ± 6.2	21.5 (12–43)	
Yes	25.5 ± 7	25 (15–37)	
**Support provided by teammates**			0.119
No.	24.4 ± 7.8	23 (12–43)	
Yes	21.3 ± 5.8	21 (12–37)	
**Feeling alone in the process**			0.521
No.	22.3 ± 7.2	21 (12–43)	
Yes	20.9 ± 5.7	21 (12–35)	
**Receiving psychological support after the process**			0.543
No.	21.7 ± 6.9	21 (12–43)	
Yes	21.5 ± 4.5	23 (12–29)	
**Application to law enforcement/judiciary**			0.268
No.	21.5 ± 6.7	20.5 (12–43)	
Yes	22.4 ± 6	23 (12–37)	

Values are presented as mean ± standard deviation and median (min–max). Statistical significance was determined using appropriate tests; *p* < 0.05 was considered significant

As shown in Table 3, female surgical nurses reported significantly higher total scores (18.3 ± 6.9) than male surgical nurses (15.8 ± 4.7; *p* = 0.010), indicating a greater perception or experience of sexual harassment. Surgical nurses with a high school or associate degree scored higher (19.4 ± 6.7) than those with a bachelor’s degree (17.0 ± 6.1; *p* = 0.036), suggesting an association between educational level and sensitivity or exposure. Married surgical nurses had higher scores than single surgical nurses (18.2 ± 6.1 vs. 17.1 ± 6.9; *p* = 0.031).

Surgical nurses with a history of prior violence exposure reported significantly higher scores (20.8 ± 6.5) than those without such exposure (14.1 ± 4.3; *p* < 0.001). Similarly, those who experienced sexual violence had notably elevated scores (24.3 ± 4.9) compared to those who had not (17.5 ± 6.1; *p* < 0.001). Interestingly, those not exposed to physical violence reported higher scores (21.9 ± 6.2) than those who were exposed (18.6 ± 6.6; *p* = 0.002), which may reflect differences in perception or coping mechanisms.

Surgical nurses who had experienced domestic violence had lower scores (16.1 ± 5.1) compared to those who had not (21.6 ± 6.4; *p* < 0.001). Exposure to violence by relatives or friends (24.6 ± 9.3) and by patients or their relatives (22.1 ± 5.7) was also associated with significantly higher perception scores compared to those who were not exposed (*p* = 0.034 and *p* = 0.003, respectively).

As shown in Table 4, significant differences were observed in the observed sexual harassment subscale scores based on gender, education level, marital status, prior exposure to violence, experience of sexual violence, and exposure to violence by family members, colleagues, and patients or their relatives, as well as responses such as distancing from the perpetrator and teammate support (*p* < 0.05).

**Table 4:** Comparison of observed sexual harassment sub-dimension scores by demographic and exposure variables

Descriptive Characteristics and Questions on Violence	Observed Sexual Harassment		*p*-value
	Mean ± SD	Median (Min-Max)	
**Gender**			**0.052**
Male	**3.2 ± 1.3**	**3 (2–6)**	
Woman	**3.7 ± 1.8**	**4 (2–11)**	
**Education level**			**0.010**
High School/Associate Degree	**4.2 ± 1.8**	**4 (2–8)**	
Bachelor’s degree	**3.3 ± 1.6**	**3 (2–11)**	
Master’s degree	**4 ± 2**	**4 (2–10)**	
PhD	**4.1 ± 1.3**	**4 (2–6)**	
**Marital status**			**0.022**
Single	**3.4 ± 1.7**	**2.5 (2–10)**	
Married	**3.8 ± 1.7**	**4 (2–11)**	
**Previous exposure to violence**			**< 0.001**
Not exposed to violence	**2.7 ± 1.3**	**2 (2–9)**	
Subjected to violence	**4.3 ± 1.7**	**4 (2–11)**	
**Sexual violence**			**< 0.001**
Not exposed to violence	**3.7 ± 1.6**	**4 (2–8)**	
Subjected to violence	**4.9 ± 1.5**	**5 (2–11)**	
**Perpetrator - family (mother/father/sibling)**			**0.009**
Not exposed to violence	**4.5 ± 1.6**	**4 (2–11)**	
Subjected to violence	**3.4 ± 1.8**	**2 (2–7)**	
**Perpetrator - work colleague**			**0.028**
Not exposed to violence	**4.2 ± 1.7**	**4 (2–11)**	
Subjected to violence	**4.8 ± 1.6**	**5 (2–8)**	
**Perpetrator of violence - patient/patient relatives**			**0.029**
Not exposed to violence	**3.9 ± 1.8**	**4 (2–10)**	
Subjected to violence	**4.6 ± 1.5**	**4 (2–11)**	
**Supporting distancing from the harassing environment/person**			**0.002**
No.	**4.3 ± 1.6**	**4 (2–11)**	
Yes	**5.7 ± 1.3**	**6 (4–8)**	
**Support provided by teammates**			**0.045**
No.	**5.3 ± 1.9**	**5 (2–11)**	
Yes	**4.4 ± 1.4**	**4 (2–8)**	

Values are presented as Mean ± SD and Median (Min–Max). SD: Standard Deviation; Min: Minimum; Max: Maximum

Although women had higher mean scores (3.7 ± 1.8) compared to men (3.2 ± 1.3), this difference was marginally non-significant (*p* = 0.052). Surgical nurses with a high school or associate degree reported the highest scores (4.2 ± 1.8), whereas those with a bachelor’s degree had the lowest (3.3 ± 1.6; *p* < 0.05). Married surgical nurses scored significantly higher than single surgical nurses (3.8 ± 1.7 vs. 3.4 ± 1.7; *p* = 0.022).

Those with prior exposure to violence had significantly higher scores (4.3 ± 1.7) than those without such exposure (2.7 ± 1.3; *p* < 0.001). Similarly, surgical nurses who had experienced sexual violence reported higher scores than those who had not (4.9 ± 1.5 vs. 3.7 ± 1.6; *p* < 0.001). In contrast, exposure to domestic violence was associated with lower scores (3.4 ± 1.8 vs. 4.5 ± 1.6; *p* = 0.009).

Exposure to violence by colleagues (4.8 ± 1.6 vs. 4.2 ± 1.7; *p* = 0.028) and patients or their relatives (4.6 ± 1.5 vs. 3.9 ± 1.8; *p* = 0.029) was also associated with significantly higher scores. Surgical nurses who supported distancing from the perpetrator had higher scores (5.7 ± 1.3) than those who did not (4.3 ± 1.6; *p* = 0.002). Notably, surgical nurses who did not receive teammate support reported higher scores (5.3 ± 1.9) than those who did (4.4 ± 1.4; *p* = 0.045), suggesting a potential protective role of team support (Table 4).

As presented in Table 5, verbal sexual harassment scores differed significantly by gender, marital status, previous exposure to violence, exposure to sexual and physical violence, and violence perpetrated by family members, relatives or friends, and patients or their relatives (*p* < 0.05).

**Table 5:** Comparative analyses by the verbal sexual harassment sub-dimension scores among healthcare workers

Descriptive Characteristics and Violence-Related Questions	Verbal Sexual Harassment		*p*-value
	Mean ± SD	Median (Min–Max)	
**Gender**			**0.001**
Male	**8 ± 2.8**	**6 (6–18)**	
Female	**9.6 ± 4**	**8 (6–23)**	
**Marital Status**			**0.039**
Single	**9 ± 4.1**	**6 (6–22)**	
Married	**9.4 ± 3.5**	**8.5 (6–23)**	
**Previous Exposure to Violence**			**< 0.001**
Not exposed	**7.2 ± 2.6**	**6 (6–21)**	
Exposed	**11 ± 3.8**	**12 (6–23)**	
**Exposure to Sexual Violence**			**< 0.001**
Not exposed	**9 ± 3.7**	**7 (6–22)**	
Exposed	**13.1 ± 2.6**	**12 (6–23)**	
**Exposure to Physical Violence**			**< 0.001**
Not exposed	**11.7 ± 3.6**	**12 (6–23)**	
Exposed	**9.5 ± 3.9**	**8 (6–20)**	
**Perpetrator – Family (Parent/Sibling)**			**< 0.001**
Not exposed	**11.4 ± 3.7**	**12 (6–23)**	
Exposed	**8.1 ± 3.1**	**6 (6–15)**	
**Perpetrator – Relative/Friend**			**0.028**
Not exposed	**10.7 ± 3.5**	**12 (6–23)**	
Exposed	**13.3 ± 5.2**	**14 (6–22)**	
**Perpetrator – Patient/Patient’s Relative**			**< 0.001**
Not exposed	**9.5 ± 3.9**	**7.5 (6–20)**	
Exposed	**11.9 ± 3.4**	**12 (6–23)**	

Values are presented as mean ± standard deviation and median (min–max). Verbal sexual harassment sub-dimension scores were compared across sociodemographic and violence-related characteristics. Statistically significant differences are indicated (*p* < 0.05)

SD: Standard Deviation, Min: Minimum, Max: Maximum

Female surgical nurses reported significantly higher scores (9.6 ± 4.0) than males (8.0 ± 2.8; *p* = 0.001). Similarly, married individuals had higher scores (9.4 ± 3.5) compared to single individuals (9.0 ± 4.1; *p* = 0.039), suggesting potential differences in perception or exposure based on marital status.

Surgical nurses with prior exposure to violence reported higher scores (11.0 ± 3.8) than those without such exposure (7.2 ± 2.6; *p* < 0.001), and those who had experienced sexual violence had significantly higher scores (13.1 ± 2.6) than those who had not (9.0 ± 3.7; *p* < 0.001).

Interestingly, those not exposed to physical violence had higher scores (11.7 ± 3.6) compared to those exposed (9.5 ± 3.9; *p* < 0.001). Surgical nurses without a history of domestic violence also reported higher scores (11.4 ± 3.7) than those exposed (8.1 ± 3.1; *p* < 0.001). Exposure to violence from relatives or friends was associated with elevated scores (13.3 ± 5.2 vs. 10.7 ± 3.5; *p* = 0.028), as was exposure to violence by patients or their relatives (11.9 ± 3.4 vs. 9.5 ± 3.9; *p* < 0.001), underscoring the occupational vulnerability of healthcare professionals (Table 5).

A statistically significant difference was found in the physical sexual harassment scores, as presented in Table 6, based on marital status, prior violence exposure, sexual and psychological violence exposure, violence by family members (mother/father/sibling), reporting to administrators in writing, initiating legal processes, and reporting to law enforcement officers (*p* < 0.05).

**Table 6:** Comparative analyses of physical sexual harassment scores among healthcare workers

Descriptive Characteristics and Violence-Related Questions	Physical Sexual Harassment		*p*-value
	Mean ± SD	Median (Min–Max)	
**Marital Status**			**0.035**
Single	**4.8 ± 1.9**	**4 (4–18)**	
Married	**5 ± 1.6**	**4 (4–12)**	
**Previous Exposure to Violence**			**< 0.001**
Not exposed	**4.2 ± 1**	**4 (4–12)**	
Exposed	**5.5 ± 2.1**	**4 (4–18)**	
**Exposure to Sexual Violence**			**< 0.001**
Not exposed	**4.8 ± 2**	**4 (4–18)**	
Exposed	**6.3 ± 1.9**	**7 (4–12)**	
**Exposure to Psychological Violence**			**0.041**
Not exposed	**5.8 ± 2.2**	**5 (4–18)**	
Exposed	**5.1 ± 1.8**	**4 (4–12)**	
**Perpetrator – Family (Parent/Sibling)**			**0.034**
Not exposed	**5.7 ± 2.2**	**4 (4–18)**	
Exposed	**4.6 ± 1**	**4 (4–8)**	
**Reporting the Incident in Writing**			**0.037**
Not reported	**5.4 ± 1.8**	**4 (4–10)**	
Reported	**6.5 ± 2.9**	**6 (4–18)**	
**Initiation of Legal Process by the Manager After Reporting**			**0.011**
No	**5.5 ± 2.4**	**4 (4–18)**	
Yes	**6.4 ± 2.1**	**7 (4–12)**	
**Filing a Complaint to Law Enforcement/Judicial Authorities**			**0.029**
No	**5.5 ± 2.2**	**4 (4–18)**	
Yes	**6.4 ± 2.1**	**7 (4–12)**	

Values are presented as mean ± standard deviation (SD) and median (minimum–maximum). SD: Standard Deviation, Min: Minimum, Max: Maximum

As shown in Table 6, married individuals reported significantly higher physical sexual harassment scores (5.0 ± 1.6) compared to single individuals (4.8 ± 1.9; *p* = 0.035), suggesting potential differences in perception or exposure. Surgical nurses with a history of prior violence had higher scores (5.5 ± 2.1) than those without such a history (4.2 ± 1.0; *p* < 0.001). Similarly, individuals exposed to sexual violence reported significantly higher scores (6.3 ± 1.9) than those not exposed (4.8 ± 2.0; *p* < 0.001).

In contrast, those exposed to psychological violence had lower scores (5.1 ± 1.8) than those not exposed (5.8 ± 2.2; *p* = 0.041), and individuals exposed to domestic violence also reported lower scores (4.6 ± 1.0) than those not exposed (5.7 ± 2.2; *p* = 0.034), indicating differences in perceived or experienced physical sexual harassment.

Surgical nurses who reported the incident in writing had higher scores (6.5 ± 2.9) than those who did not (5.4 ± 1.8; *p* = 0.037). Those who initiated legal proceedings also had significantly higher scores (6.4 ± 2.1) compared to those who did not (5.5 ± 2.4; *p* = 0.011). Additionally, individuals who applied to law enforcement authorities reported higher scores (6.4 ± 2.1) than those who did not (5.5 ± 2.2; *p* = 0.029), suggesting the possible influence of formal reporting and legal action on perceptions of physical sexual harassment.

## Discussion

According to the 2023 Report on Violence in Healthcare by the İmdat Association, 457 incidents of violence against healthcare professionals were recorded in Türkiye between January 1 and December 20, 2023. Among the victims, 43.3% were nurses, 40.9% were physicians, and 15.8% were other healthcare personnel. Notably, 60.4% of these incidents were perpetrated by patients’ relatives, and 80.5% of the victims initiated legal action [24].

Violence and sexual harassment remain persistent challenges in healthcare settings, particularly within nursing, where close physical interaction and emotional labor are integral to professional practice. In the present research, 53.6% of nurses reported having experienced violence, and 48.1% indicated exposure to sexual violence, with sexual violence emerging as the most frequent type of abuse. These rates are substantially higher than the 26.9% prevalence reported by Jafree [25], yet al.ign with previous evidence suggesting that nurses are disproportionately affected by workplace violence, especially of a sexual nature.

Such discrepancies across studies cannot be attributed solely to methodological variations. Cultural and societal dynamics in Türkiye play a critical role in shaping attitudes toward the reporting of sexual harassment. The patriarchal social order, the association of sexuality with shame and privacy, and the fear of stigmatization or professional repercussions often inhibit disclosure. However, the online and anonymous format of data collection in this study may have alleviated these barriers, allowing nurses to express their experiences more openly and revealing higher prevalence rates than previously documented.

The predominance of women in the nursing workforce and the high level of physical proximity required in surgical care settings create structural conditions that heighten vulnerability to sexual harassment through the intersection of gender and professional roles. These findings, therefore, should not be interpreted as isolated incidents but as manifestations of broader institutional hierarchies, patriarchal norms, and cultural silence that continue to obscure and normalize sexual harassment within healthcare environments.

It was determined that nurses were predominantly exposed to violence perpetrated by patients and their relatives. Similarly, Esen and Aykal (2020) reported that 60.7% of violence against healthcare professionals was committed by patients [26]. These results indicate that the patient–healthcare worker relationship, characterized by emotional intensity and power imbalance, represents a significant risk factor for violence. In particular, patients’ experiences of pain, anxiety, and loss of control in surgical units may create conditions that facilitate aggressive behaviors.

As noted in the systematic review by Kahsay and Negarandeh (2020), sexual harassment toward female nurses persists not only in physical but also in verbal, non-verbal, and psychological forms [27]. This finding demonstrates that gender roles and patriarchal values remain influential within healthcare services. In Türkiye, where the nursing profession is predominantly female, women are often associated with socially expected roles such as being “helpful,” “patient,” and “quiet,” both professionally and culturally. Such cultural perceptions may reinforce nurses’ hesitancy to express boundaries or report harassment.

Likewise, Demirci (2018) investigated the perpetrators of violence in the healthcare sector and found that patients were the main source of verbal violence against physicians, while physicians were the primary perpetrators of sexual violence against nurses [28]. Similar findings were observed in a study conducted in Africa, where patients and their relatives, coworkers, and superiors were identified as the most common perpetrators of violence. The study also concluded that physicians were the leading source of sexual violence against nurses [29]. These differences may be attributed to variations in clinical settings, particularly those involving intensive physical and verbal interactions with patients and their families. Moreover, hierarchical power structures within healthcare institutions may further facilitate harassment behaviors. The normalization of inappropriate conduct by individuals in higher professional positions, such as physicians, often perpetuates institutional silence. Taken together, these findings suggest that both the asymmetric power dynamics in nurse–patient relationships and intra-institutional hierarchies function as key cultural and structural determinants of sexual harassment incidents toward nurses.

Health institutions, due to the requirement for physical contact with patients and the discussion of bodily functions, may inadvertently create environments where sexual impulses are stimulated. Additionally, gender imbalances in these settings often reinforce gender-based discrimination, positioning women as submissive and nurturing, while portraying men as dominant. Women are frequently stereotyped as caring, passive, or sexually attractive.

According to the 2015 Research on Domestic Violence Against Women in Turkey, conducted by the Ministry of Family and Social Policies, 3% of women aged over 15 reported experiencing sexual violence by non-partners, and 9% reported being sexually abused before the age of 15 [30]. In a retrospective multicenter study analyzing 360 White Code incident reports, it was found that the majority of healthcare workers reported as perpetrators (51.1%) were women [31]. This finding suggests a need to better understand the dynamics and reporting patterns within institutional responses to workplace violence.

In the United States, over a span of 18 years, more than six million children have been subjected to violence by family members. Additionally, women have been reported as frequent victims of male-perpetrated violence in both domestic and professional settings [32].

A literature review by Chakraborty et al. (2022), covering studies from 2010 to 2020, revealed that female healthcare workers are disproportionately exposed to violence compared to their male counterparts [33]. This result is supported by a study conducted by George et al. (2020), which found that women in healthcare experience more violent incidents than men, primarily due to gender discrimination [34].

Many studies have emphasized that female nurses are the primary victims of sexual harassment in healthcare settings. However, contrasting findings were reported in a cross-sectional study by Jeong and Chang (2022) conducted in South Korea, which provided compelling evidence that male nurses working within a predominantly female profession also face sexual harassment. According to the study, 65.2% of male nurses reported experiencing sexual harassment at least once during their professional careers, with an average of 3.2 incidents per person. The only significant factor associated with the occurrence of sexual harassment was the nurses’ perception of their work environment. Male nurses who viewed their work environment negatively reported higher rates of harassment. These findings suggest that male nurses are also vulnerable to sexual harassment, regardless of gender, challenging traditional assumptions about gender-specific victimization. The authors emphasize the need for clear institutional policies, safe and confidential reporting mechanisms, and targeted training and support programs to improve the overall work environment in nursing [35].

Sexual violence represents one of the most psychologically and socially destructive forms of abuse encountered by nurses. It not only threatens personal safety but also leads to feelings of fear, shame, and isolation that may deeply affect professional functioning and self-perception. In this research, 50.8% of nurses reported experiencing sexual violence and feeling isolated during the process. Unlike other types of violence, sexual harassment often evokes fear of rape, sexual dysfunction, and internalized guilt. Consistent with these results, Oğan and Sercan [36] reported that employees who experienced workplace violence felt unsafe, perceived their institutions as unprotective, and suffered from loneliness and helplessness.

In the current findings, only 24.0% of nurses reported receiving support following sexual harassment, and 29.3% stated that they had reported the incident to law enforcement authorities. Similarly, Yılmaz (2020) found that 39.6% of healthcare workers filed complaints after physical violence, whereas only 22.5% did so following sexual violence, illustrating a general reluctance to report such incidents [37]. In Türkiye, the circular titled “Investigation of Crimes Committed Against Healthcare Professionals,” issued by the Ministry of Interior, aims to prevent all forms of violence against healthcare workers and outlines measures involving collaboration among hospital police, law enforcement officers, private security personnel, and hospital staff. According to the circular, filing a complaint by the victim is not a prerequisite for initiating legal proceedings. Law enforcement officers, particularly hospital police stationed in healthcare institutions, are required to act immediately in the event of such incidents, inform the public prosecutor, and initiate the necessary investigation procedures. However, the findings of the present study indicate that, in practice, legal actions are predominantly initiated upon the victim’s complaint. This suggests that the provisions stipulated in the circular are not fully implemented in practice, and that the legal regulations designed to protect healthcare professionals in Türkiye remain limited in their effectiveness [38].

These patterns reflect the persistent silence surrounding sexual harassment within healthcare institutions and the cultural barriers that discourage reporting. Deeply rooted patriarchal norms and the fear of stigmatization continue to shape women’s responses to such experiences, fostering resignation and self-blame. The ineffectiveness of institutional mechanisms further aggravates the issue. Ergen (2022) found that nearly all surgical nurses perceived the institutional White Code system as slow and ineffective; 68.2% believed that reporting would bring no benefit, and 59.8% described the legal procedures as overly complicated [39]. Similarly, Kaynak (2019) reported that although 26.7% of employees activated the White Code system, no action was taken in nearly half of the cases (49.1%), while 63.9% did not take any action after experiencing violence [40].

Overall, the findings suggest that sexual violence has deeper psychosocial consequences for nurses than other forms of violence and that existing institutional support and reporting systems remain insufficient. This situation highlights the urgent need to strengthen organizational policies, simplify reporting procedures, and foster a workplace culture that values transparency, psychological safety, and gender equality.

In this study, 40.7% of nurses reported incidents of harassment to their managers 61% verbally and 30.1% in writing. This pattern indicates notable inconsistencies in reporting pathways and suggests that both social norms and organizational culture significantly influence how sexual harassment is perceived and addressed. Environmental factors, including managerial and peer support, further demonstrate that nurses’ attitudes toward reporting are shaped less by individual decision-making and more by institutional and sociocultural dynamics.

Consistent with these findings, Ünlüsoy Dinçer reported that 97.0% of nurses who experienced sexual harassment stated that no action was taken against the perpetrator, with only one respondent noting that the perpetrator’s caregiving responsibilities were suspended. Similarly, among nurses exposed to mobbing, 91.7% indicated that no measures were taken; only 0.9% mentioned the initiation of legal proceedings or dismissal, and 4.6% reported a verbal warning [41].

Lee et al. (2024) conducted a large-scale cross-sectional secondary analysis in British Columbia, Canada, involving 4,109 nurses, to examine workplace violence reporting behaviours and reasons for underreporting among nurses. The study categorized sources of violence as *Type II* (patients and visitors) and *Type III* (coworkers), and explored five forms of aggression: physical assault, threats of assault, emotional abuse, verbal sexual harassment, and sexual assault. The findings revealed that nurses were less likely to formally or informally report incidents involving threats, emotional abuse, or verbal sexual harassment compared with physical violence. The most frequently cited reason for not reporting was the perception that “reporting would not change anything.” Furthermore, nurses who perceived their institutions as committed to violence prevention were significantly more likely to report such incidents. The authors emphasized that healthcare organizations must establish reporting systems that are safe, accessible, and supported by leadership, thereby reducing fear of reprisal and enhancing institutional accountability [42].

In the study conducted by Song et al., it was reported that the most significant reason nurses did not report incidents of violence was their lack of awareness regarding how and which types of violence should be reported. The second major barrier to reporting was found to be related to organizational culture and attitudes toward violence. Furthermore, 50.6% of participants stated that, following a report, more attention was given to patients than to healthcare workers; 38.5% believed there was insufficient supervisory support, and 21.8% felt that reporting the incident would not lead to any change [43].

These results reveal how hierarchical power structures, patriarchal management practices, and a pervasive culture of institutional silence contribute to the concealment and normalization of sexual harassment in healthcare settings. In this research, higher scores of physical sexual abuse were associated with more serious legal actions and written reporting, suggesting that the tangible nature of physical abuse may compel victims to pursue justice more frequently than in cases of verbal or non-contact harassment. Nonetheless, the overall rate of reporting such incidents to law enforcement remains low, likely reflecting a widespread belief among healthcare professionals that legal procedures are ineffective or fail to deliver justice.

Taken together, these findings illustrate that nurses’ responses to sexual harassment are not solely the product of individual perceptions or experiences but are deeply shaped by institutional hierarchies, gendered power relations, and culturally reinforced silence surrounding sexual violence in professional environments.

## Conclusion

This study revealed a high prevalence of sexual violence experienced by nurses working in hospital settings, particularly within surgical clinics. Based on the findings, the following recommendations are proposed to prevent such incidents and improve institutional responses:

- Educational programs at the undergraduate, postgraduate, and continuing education levels should be developed to enhance nurses’ awareness of their legal rights and professional responsibilities concerning sexual harassment.
- Comprehensive reporting systems and internal regulations addressing intimidation, as well as physical, sexual, and verbal violence, should be established in collaboration with nursing staff.
- Hospital administrations must provide consistent support to nurses throughout incidents of sexual violence, including legal follow-up and psychological assistance.

This study aims to contribute to the growing body of scientific knowledge on sexual harassment in healthcare settings and to inform future research on this subject. It is further recommended that similar studies be conducted with larger and more diverse samples across various healthcare settings to improve generalizability within Türkiye.

### Limitations

The findings should be interpreted in consideration of the study’s methodological approach and sample characteristics. The use of an online self-reported questionnaire may have introduced potential biases such as social desirability or recall effects. The cross-sectional design limits causal inference, while the restriction to surgical nurses constrains the generalizability of the results. Given the sensitivity of the topic, some participants may have refrained from disclosing their experiences. Nevertheless, the results offer meaningful insights into surgical nurses’ perceptions of sexual harassment and provide a valuable basis for future research in this field.

## Supplementary Information

Below is the link to the electronic supplementary material.

Supplementary Material 1

Supplementary Material 2

## Data Availability

The datasets used and/or analyzed during the current study are available from the corresponding author on reasonable request.

## Abbreviations

WSH: Workplace sexual harassment
G*Power: General Power Analysis Program
SD: Standard deviation
Min: Minimum
Max: Maximum
N: Total sample size
n: Subgroup sample size
%: Percentage
