# Supplementary material for: Surgical nurses’ perceptions of experienced sexual harassment behaviors
Source: BMC Nurs. 2025 Dec 9;25:35. doi: 10.1186/s12912-025-04202-6 (PMC12801705; doi:10.1186/s12912-025-04202-6)
Supplement: Supplementary file 1 — Supplementary Material 1 [file 12912_2025_4202_MOESM1_ESM.docx]

Attitudes and Behaviours Towards Sexual Harassment Form

1. Previous exposure to violence *

Select only one option.


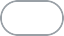
 Yes
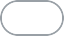
 No

2. If exposed, type of violence

Select all that apply.


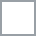
 Physical
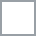
 Sexual


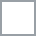
 Psychological
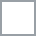
 Economic

3. If you have been exposed to violence before, who perpetrated it?

Check all that apply.


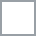
 Spouse/Partner

Friend/Relative
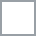
 Mother/Father/Sibling


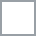


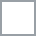
 Work Colleague


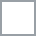
 Manager


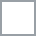
 Patinet/Patient’s Relative

4. If you have been exposed to sexual harassment at work, have you reported this to your manager?

Select all that apply.


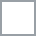
 I did not report it
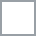
 I report it verbally
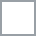
 I report it writing

5. If you reported it, did your manager initiate legal proceedings regarding the situation?

Select only one option.


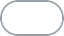
 Yes
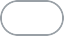
 No

6. Did your manager support you?

Select all that apply

.
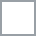
My manager supported me regarding this matter.
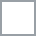
 Provided psychological support.


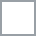
Effectively followed up on the legal process.

Supported my removal from the harassing environment/person. (E.g., reassignment to a different department/having other nurses take care of the patient.)


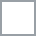


Did not provide any support


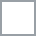


7. Did your team members support you?

Select only one option.


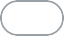
 Yes
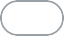
 No

8. Did you feel alone during this process?

Select only one option.


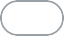
 Yes
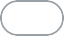
 No

9. Did you receive psychological support after the process?

Select only one option.


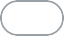
 Yes
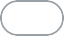
 No
