# Supplementary material for: Surgical nurses’ perceptions of experienced sexual harassment behaviors
Source: BMC Nurs. 2025 Dec 9;25:35. doi: 10.1186/s12912-025-04202-6 (PMC12801705; doi:10.1186/s12912-025-04202-6)
Supplement: Supplementary file 2 — Supplementary Material 2 [file 12912_2025_4202_MOESM2_ESM.docx]

Introductory Characteristics Form

1. Age*:

2.Gender *

Please select only one option.


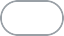
 Female
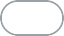
 Male

3.Your educational status*

Please select only one option.


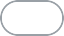
 High school/Associate degree
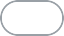
 Bachelor's degree


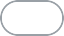
 Master's degree
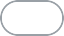
 Doctorate

4.Marital status *

Please select only one option.


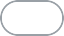
 Single
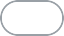
 Married

5. Number of children *

Please select only one option.


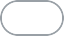
 1


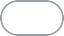
 2


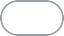
 3

Other:

6. Unit you work in *

7. Length of time working in your profession *

8. Daily working hours *
